# P-1275. Quasi-experimental Approaches for Estimating Epidemiological Efficacy of Non-randomised Field Trials: Applications to Wolbachia Interventions

**DOI:** 10.1093/ofid/ofae631.1456

**Published:** 2025-01-29

**Authors:** Jo Yi Chow, Geng Lin, Somya Bansal, Ary Hoffmann, Lee Ching Ng, Jue Tao Lim

**Affiliations:** Nanyang Technological University, Singapore, Singapore; Nanyang Technological University, Singapore, Singapore; National University of Singapore, Singapore, Not Applicable, Singapore; University of Melbourne, Melbourne, Victoria, Australia; National Environment Agency, Singapore, Not Applicable, Singapore; LKCMedicine, NTU, Singapore, Singapore

## Abstract

**Background:**

*Wolbachia* symbiosis in *Aedes aegypti* is an emerging biocontrol measure against dengue. However, assessing its real-world efficacy is challenging due to the non-randomised, field-based nature of most intervention studies. This research re-evaluates the spatial-temporal impact of *Wolbachia* interventions on dengue incidence using a large battery of quasi-experimental methods and assesses each method’s validity.

Aggregated intervention efficacies by event time for each study site

**Figure:**
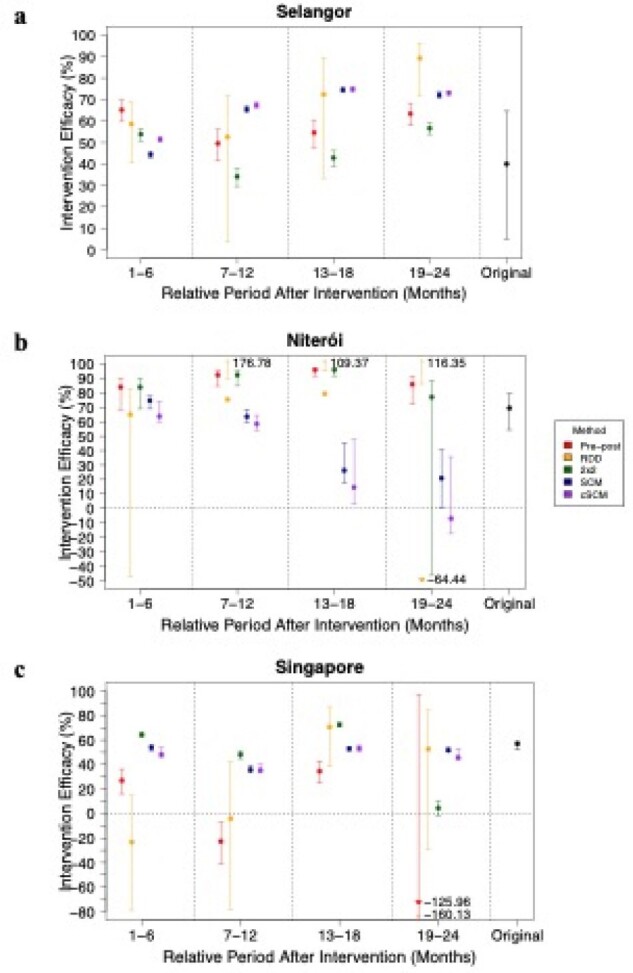

Aggregated intervention efficacies for (a) Selangor, (b) Niterói, and (c) Singapore based on event time. Each efficacy assessment method is represented by a specific color: red for Pre-post, yellow for RDD (Regression Discontinuity Design), green for 2x2 DiD (Difference-in-Differences), blue for SCM (Synthetic Control Method), and purple for cSCM (count Synthetic Control Method). The intervention efficacy (IE) reported in the original paper is depicted on the right side of each diagram, represented by the colour black. The dot represents the IE point estimate for each method, while the line indicates the corresponding confidence intervals. Extreme confidence interval values and point estimates are denoted by arrows, with their respective values displayed next to the arrow.

**Methods:**

A systematic search for *Wolbachia* intervention data was conducted via PUBMED. Efficacy was reassessed using commonly-used quasi-experimental approaches with extensive robustness checks, including geospatial placebo tests and a simulation study. Intervention efficacies across multiple study sites were computed using high-resolution aggregations to examine heterogeneities across sites and study periods. We further designed a stochastic simulation framework to assess the methods’ ability to estimate intervention efficacies (IE).

Aggregate intervention efficacy (IE) estimates (%) of Wolbachia releases on total dengue incidence rates across all intervention sites

**Table 2:**
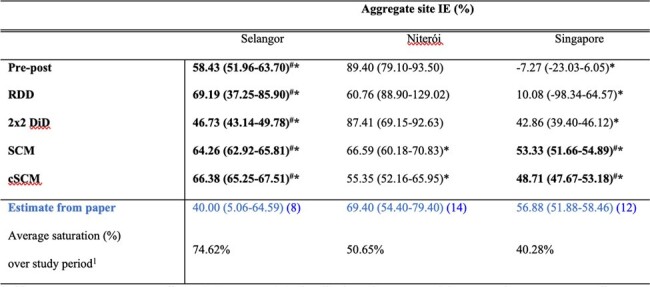
Aggregate intervention efficacy (IE) estimates (%) of Wolbachia releases on total dengue incidence rates across all intervention sites. The estimates are reported for each method and are accompanied by the corresponding confidence intervals (in parentheses). The IE estimate reported in the respective original papers, highlighted in blue, is provided for comparison. Numbers in parenthesis represent lower and upper bounds for 95% confidence intervals, estimated using the bootstrapping procedure. # indicates that the estimate passed in-time placebo checks while * indicates that it passed in-space placebo check. Bolded figures represent significant IE estimates which also passed in-space and in-time placebo checks.

**Results:**

*Wolbachia* interventions in Singapore, Malaysia, and Brazil significantly decreased dengue incidence, with reductions ranging from 48.17% to 69.19%. IEs varied with location and duration. Selangor showed increasing efficacy over time, while Niterói exhibited initial success with subsequent decline, hinting at operational challenges. Singapore's strategy was highly effective despite partial saturation. Simulations identified Synthetic Control Methods (SCM) and its variant, count Synthetic Control Method (cSCM), as superior in precision, with the smallest percentage errors in efficacy estimation. These methods also demonstrated robustness in placebo tests.

**Conclusion:**

*Wolbachia* interventions exhibit consistent protective effects against dengue. SCM and cSCM provided the most precise and robust estimates of IEs, validated across simulated and real-world settings.

**Disclosures:**

**All Authors**: No reported disclosures

